# Emotional and behavioural problems in Swedish preschool children rated by preschool teachers with the Strengths and Difficulties Questionnaire (SDQ)

**DOI:** 10.1186/s12887-017-0864-2

**Published:** 2017-04-21

**Authors:** Berit M. Gustafsson, Marie Proczkowska-Björklund, Per A. Gustafsson

**Affiliations:** 1 Department of Clinical and Experimental Medicine, Center for Social and Affective Neuroscience, Linköping University, Linköping, Sweden; 2 Division of Psychiatrics & Rehabilitation/Region, Child Psychiatric Clinic, Högland Hospital, Jönköping, Sweden; 3 Department of Child and Adolescent Psychiatry, Linköping University, Linköping, Sweden; 4 Division of Psychiatrics & Rehabilitation/Jönköping County, Psychiatric Clinic, Hospital of Jönköping, Jönköping, Sweden; 5 CHILD research environment, Jönköping University, Jönköping, Sweden; 6 Division of Psychiatrics & Rehabilitation/Region Jönköping County, Child Psychiatric Clinic, Högland Hospital, Skansgatan 9, 571 37 Nässjö, Sweden

**Keywords:** Preschool children, Behaviour problems, Mental health, Emotional distress, Strengths and difficulties questionnaire, Screening - longitudinal study

## Abstract

**Background:**

There is a high risk that young children who show early signs of mental health problems develop symptoms in the same or overlapping areas some years later. The Strengths and Difficulties Questionnaire (SDQ) is widely used to screen externalizing and internalizing problems early in life. In Sweden 80–90% of all children aged 1–5 years go to preschool and preschool is thus an appropriate context for finding early signs of mental health problems among children.

**Methods:**

This study is part of a longitudinal project too investigate the frequency of emotional and behavioural problems for children between 1 and 5 years of age in Sweden. The SDQ including the impairment supplement questions were rated by preschool teachers too establish Swedish norms for SDQ in preschool children.

**Results:**

The sample involved 815 children with a mean age of 42 months (SD = 16, range 13–71 months). 195 children were followed longitudinally for three years. There were significant differences between boys and girls on all subscales except for the Emotional subscale. The prevalence of behavioural problems was similar to other that in European countries, except for Prosocial behaviour, which was rated lower, and Conduct problems, rated higher. Swedish children were estimated to have more problems in the preschool setting, scored by preschool teachers. The development of behaviour over time differed for the different subscales of SDQ.

**Conclusions:**

The teacher version of the SDQ, for 2–4 year-olds, can be used as a screening instrument to identify early signs of emotional distress/behavioural problems in young children. Preschool teachers seem to be able to identify children with problematic behaviour with the use of SDQ at an early age. The development of behaviour over time differs for the different subscales of SDQ. The Swedish norms for SDQ are to a large extent, similar to findings from other European countries.

## Background

Mental health problems can be seen in all age groups and are a major factor in societal costs [1, 2]. There is a high risk that children (under the age of 5) who show early signs of mental health problems/behaviours develop symptoms in the same or overlapping areas while growing up and these problems may be long term [3].

Identifying mental health problems in children may be a starting point both for early intervention and planning for those who need support in a longer perspective. Detecting early signs of mental health problems and identifying high-risk groups for pro-active intervention with the aim of preventing health problems later in life would be desirable [4, 5].

Mental health problems in young children can be “under the threshold” for what is considered to be sufficiently severe to be a disorder or to be considered to be a diagnosis according to International Statistical Classification of Diseases and Related Health Problems (ICD) [6] or Diagnostic and Statistical Manual of Mental Disorders (DSM) [7]. But the child may still exhibit problems with functioning and/or limitations in functioning in everyday life.

Many studies describe the frequency of psychiatric disorders in school-age children, adolescents and adults but there are fewer studies concerning preschool children [5, 8–10]. Prevalence studies using the Strengths and Difficulties Questionnaire (SDQ) have shown functionally impairing psychopathology in 10–15% of 5–15 years old children and adolescents [11, 12]. Other studies using different screening instruments have found that over 25% of children and adolescents show signs of psychiatric disorders, yielding prevalence figures for emotional and behavioural disorders for preschool children ranging from 14% to 26% [9, 13–15]. However, the prevalence rates are substantially lower when using diagnostic criteria such as attention-deficit hyperactivity disorder (ADHD); 3.3%, oppositional defiant disorder (ODD); 6.6%, conduct disorder (CD); 3.3%, depression; 2.1%, separation anxiety disorder (SAD); 2.4% or generalized anxiety disorder (GAD); according to DSM-IV [8]. In earlier studies, relatively lower frequencies of problems as measured by SDQ have been found in children from Nordic countries in contrast to other industrialized countries [16–18].

In Sweden, 80–90% of all children aged 1–5 years attend preschool. Preschool staffs have a documented high level of understanding concerning child development. The preschool setting is accordingly an appropriate context for finding signs of mental health/behaviour problems among young children [19].

The Strengths and Difficulties Questionnaire (SDQ) is widely used in community, clinical and research settings to screen for externalizing and internalizing problems [12, 20, 21]. SDQ has been translated into many languages, and exists in 3 versions: parent, teacher and a self-rating version for older children [17, 22–24] and parent and teacher versions for children of 2–7 years of age [5, 25–27]. SDQ has been translated into Swedish and validated for parental use for children of 5–10 years old, and it has demonstrated good psychometric properties [28, 29]. The self-rated version for young people has also been validated in Sweden [30].

There are a few studies using SDQ teachers’ version for children of 1–5 years old [31, 32]. The SDQ teacher version for young children aged 1–5 years has recently been validated in Sweden [33].

## Methods

### Aim

To study the frequency of emotional and behavioural problems, as rated by preschool teachers using SDQ, for children between 1 and 5 years of age in Sweden, and establish Swedish norms for SDQ in preschool children.

### Procedure

This study is part of a longitudinal project, with three yearly waves, studying preschool children’s mental health and functioning in the preschool setting [34].

Preschools from a stratified sample of different sized Swedish municipalities, representing both large (>200,000 inhabitants), middle sized (50000–200,000 inhabitants) and small municipalities (<50,000 inhabitants) were invited to participate. The preschool managements in the various municipalities were contacted and consent was requested for participation of their preschool units. They then addressed their preschool teachers for consent. Both written and video information was made available to management, teachers and parents.

If a preschool class was to be included, at least one preschool teacher had to consent to participate. In all, 311 preschool teachers in 81 different preschool classes participated. The preschool teachers asked all parents (*n* = 3230) for written informed consent. Each preschool teacher rated SDQ for an average of two children. Answers were based of all their knowledge of the child and covered a period of at least the two preceding weeks. It took about 20–30 min to answer the entire questionnaire.

### Instruments

The Strength and Difficulties questionnaire (SDQ):

In this study, the teacher version for children aged 2–4 was used, including the impairment supplement [35].

The SDQ is a 25-item screening questionnaire. The SDQ teacher version has been shown to have satisfactory psychometric properties to identify children of 3–5 years of age with emotional and behavioural difficulties [31–33].

The items are divided into five subscales of five items, each generating scores for Emotional symptoms, Conduct problems, Hyperactivity/inattention, Peer relationship problems and Pro-social behaviours. Each item is scored on a three-point scale: not true, somewhat true; and certainly true. The four above scales (i.e., all except Prosocial behaviour) are summarized to generate a total-difficulties score. There is also an impairment supplement consisting of questions about the impact on the child’s daily life of the problem identified [35, 36].

The impairment supplement starts with the question “Overall, do you think that this child has difficulties in one or more of the following areas: emotions, concentration, behaviour or being able to get on with other people?” If the preschool teacher answers “Yes” to this question, they are asked to answer the following questions about these difficulties;” How long have these difficulties been present?” and” Do the difficulties put a burden on you or the class or group as a whole?”

The impairment supplement questions, which are included in the impact score, are: “Do the difficulties upset or distress the child”. For the question “Do the difficulties interfere with the child’s everyday life in the following areas?” the specification “Peer relationship and Learning” is substituted in the Swedish translation with the situations: “Free play, Organized situations and Routine situations”. This is an adaption to the structure in the Swedish preschool environment, which is not as “classroom-like” as in many other countries. Total impact scores were calculated according to the scoring algorithm recommended on the SDQ scoring site. However, the range is 0–8, since there is one question more in the Swedish version. Ratings of “Not at all” and “Only a little” were scored as 0, “Quite a lot” as 1 and “A great deal” as 2 [35].

#### Additional questions:

The Preschool teachers also answered questions about demographics and the questions; "Has the child a mother tongue other than Swedish" and "Is the child in need of special support”.

### Statistical methods

The data were analysed in SPSS version 23. Demographic data are presented with mean, standard deviation (SD), median and cut-offs for the 90th and 10th percentile. Correlation estimates were made using the Pearson correlation coefficient (r). The Mann Whitney U-test (two-tailed) was used for comparisons between boys and girls. For graphic longitudinal presentation we used boxplots over three years, for first and third quartiles, medians and means, split by gender.

## Results

### Participants

In this analysis, the children were included the first time they participated in the study, using all three yearly waves of data collection. In the first data collection, 1615 children were invited to participate and parents of 663 (41.6%) gave their consent. Of these, preschool teachers completed the SDQ for 651 (40%) children. In the second year, preschool teachers completed the SDQ for 91 new children, and in the third year 73 new children were included, resulting in a total of 815 children with complete SDQ forms. Of the 651 children who participated the first year, 195 (30%) children took part in the study at all three data collections. Longitudinal data of these children are presented. The participation rate and gender distribution of these longitudinally followed children were similar for children of different ages, and mother tongues other than Swedish.

### Demographics

The sample involved 815 children: 424 boys and 391 girls with a mean age of 3 and half years, or 42 months (SD = 16, range 13–71). There was no significant mean age difference between genders. Approximately 47% lived in small municipalities, 45% in medium-sized municipalities and 8% in large municipalities. In Sweden as a whole the distribution is 43% in small, 16% in medium-sized and 41% in large municipalities, i.e. large municipalities are under-represented and medium-sized municipalities are over-represented in our sample. A total of 91% of the children lived with both their biological parents, 3% had shared living and 5% lived with only their mother at the date of the investigation, 28% of the children had a mother tongue other than Swedish (i.e. were second generation immigrants). This corresponds quite well with the statistics about preschool children in Statistics Sweden (SCB) [37, 38].

In this sample the preschool group mean size was 22 children. The preschool teachers estimated that 4.3% of the children were in need of special support. Concurrent validity for the SDQ total scale was found satisfactory in an earlier study using the SDQ prosocial scale, supplementary questions in SDQ, and the total scores for Child-Teacher Report Form (C-TRF) [39] and Child Engagement Questionnaire (CEQ) [40], respectively as comparisons [33].

The sample for the longitudinal analysis involved 195 children who participated in all the three years, 110 (56%) boys and 85 (44%) girls with a mean age of 2 years and 8 months, or 32 months (SD = 9, range 15–57), the first year.

The participation rate and gender distribution were similar for children of different ages. 23.3% of the children had a mother tongue other than Swedish and 3.8% were judged to be in need of special support.

### The subscales and cut-offs

The medians, ranges, modes and cut-offs for the 90th percentile (10th percentile on Prosocial scale), were calculated for SDQ with respect to gender (see Table 1).

**Table 1:** Medians, ranges, modes, and cut-offs in accordance with 90th percentiles (10th percentile on the prosocial scale), on subscales and the total difficulties scale. There are different numbers of participants (1–5 years of age) for boys (*n*.420–424) and girls (*n*.386–391) in the different subscales. The Mann Whitney U-test (two-tailed) was used for comparisons between boys and girls

		Boys			Girls			
	Cut-off	Median	Range	Mode	Median	Range	Mode	*p*
Total Difficulties Scale (without Prosocial)	12	6	0–25	0	5	0–24	0	.003
Prosocial	3	6	0–10	10	7	0–10	10	< .001
Hyperactivity/inattention	5	2	0–10	0	2	0–10	0	< .001
Emotional symptoms	3	0	0–7	0	0	0–6	0	.153
Conduct problems	4	1	0–10	0	1	0–9	0	.017
Peer problems	4	1	0–8	0	1	0–8	0	.013

Boys were rated as having significantly (*p* = .003) more difficulties (median 6, range 0–25) compared to girls (median 5, range 0–24) on the total scale. There were significant differences (p between < .001 to .017) between the sexes in all subscales except for the emotional scale. Boys were also rated to have significantly (*p* < .001) less Prosocial skills than girls. A cut-off in accordance with the 90th percentile was applied to all subscales, except for Prosocial where the 10th percentile would identify problematic behaviour.

#### Total difficulties scale

On the Total scale, a cut-off at 11 covers 19.1% of the boys and 10.8% of the girls, 12 covers 14.9% of the boys and 8.5% of the girls, 13 covers 11.8% of the boys and 7.2% of the girls. Thus, the material favours a cut-off at 12 on the Total scale.

#### Prosocial subscale

A cut-off in accordance with the 10th percentile was aimed at, since low ratings of Prosocial skills indicate difficulties in this area. A cut-off at 2 covers 9.9% of the boys and 5.1% of the girls, 3 covers 15.1% of the boys and 10.5% of the girls, 4 covers 25.5% of the boys and 19.5% of the girls. Thus, the material favours a cut-off at 3 on the Prosocial subscale.

#### Hyperactivity subscale

A cut-off at 4 covers 22.9% of the boys and 14.7% of the girls, 5 covers 12.0% of the boys and 7.5% of the girls, 6 covers 9.4% of the boys and 5.4% of the girls. Thus, the material favours a cut-off at 5 on the Hyperactivity subscale.

#### Emotional subscale

A cut-off at 2 cover 11.4% of the boys and 22.1%of the girls, 3 covers 4.5% of the boys and 14.8% of the girls, 4 covers 2.1% of the boys and 10.2% of the girls. The material favours a cut-off at 3 on the Emotional subscale.

#### Conduct problem subscale

A cut-off at 3 covers 14.8% of the boys and 11.1% of the girls, 4 covers 10.2% of the boys and 6.2% of the girls, 5 covers 5.0% of the boys and 3.9% of the girls. Thus, the material favours a cut-off at 4 on the Conduct problem subscale.

#### Peer problem subscale

A cut-off at 3 covers 21.5% of the boys and 16.9% of the girls, 4 covers 11.3% of the boys and 9.5% of the girls, 5 covers 4.7% of the boys and 2.8% of the girls. Thus, the material favours a cut-off at 4 on Peer problem subscale.

### Endorsement rates on single items

Table 2 presents the wordings of items on the Prosocial scale and on the four problem scales, and also the endorsement rates for the different response categories. The number of missing replies at the item level ranged from 11 to 86 (1.4–10.6%).

**Table 2:** Preschool teacher reports on 815 children aged 13–71 months. Endorsement rates (%) percent of response categories for each item. *Items in italics are scored reversely*

SDQ Questions	1 year (13–23 month) n.128 (16%)			2 year (24–35 month) n.203 (25%)			3 year (36–47 month) n.170 (21%)			4 year (48–59 month) n.156 (19%)			5 year (60–71 month) n.158 (19%)		
	Not true	Some what true	Certainly true	Not true	Some what true	Certainly true	Not true	Some what true	Certainly true	Not true	Some what true	Certainly true	Not true	Some what true	Certainly true
**Prosocial items**															
1. Considerate of other people’s feelings.	10.1	68.9	21.0	6.2	39.2	54.6	3.1	38.5	58.5	2.6	29.3	68.1	1.3	30.4	68.4
4. Shares readily with other children.	26.1	65.5	8.4	12.3	61.5	26.2	5.8	50.8	43.3	6.1	39.1	54.8	1.9	33.1	64.9
9. Helpful if someone is hurt, upset of feeling ill.	24.8	64.6	10.6	11.4	41.7	47.0	6.9	35.4	57.7	4.3	28.4	67.2	2.6	29.5	67.9
17. Kind to younger children.	9.6	47.9	42.6	4.2	32.5	63.3	0.8	14.0	85.3	1.7	16.4	81.9	1.4	4.8	93.8
20. Often volunteers to help others.	49.4	46.1	4.5	17.7	53.1	29.2	7.1	40.2	52.8	6.9	36.2	56.9	4.4	30.4	65.2
**Hyperactivity items**															
2. Restless, overactive, cannot stay still for long.	75.0	21.7	3.3	65.4	26.0	8.7	70.2	18.3	11.5	77.8	15.4	6.8	77.9	17.5	4.5
10. Constantly fidgeting or squirming.	69.2	26.7	4.2	64.2	25.0	10.8	64.9	22.9	12.2	76.1	16.2	7.7	75.3	19.6	5.1
15. Easily distracted, concentration wanders.	71.5	25.2	3.3	64.2	27.6	8.1	72.5	16.0	11.5	72.6	19.7	7.7	80.9	14.6	4.5
*21. Can stop and think things out before acting.*	26.3	61.6	12.1	21.5	57.7	20.8	10.8	43.8	45.4	11.2	36.2	52.6	6.4	37.8	55.8
*25. Sees tasks through to the end, good attention span.*	14.4	63.1	22.5	14.0	47.3	38.8	10.7	30.5	58.8	2.6	28.4	69.0	5.7	22.2	72.2
**Emotional items**															
3. Often complains of headaches, stomach-aches or sickness.	97.0	3.0	0	96.6	2.3	0.8	87.8	12.2	0.0	95.7	4.3	0.0	94.1	5.2	0.7
8. Many Worries, often seems worried.	86.4	12.7	0.9	90.9	8.3	0.8	79.2	16.9	3.8	82.1	16.2	1.7	78.6	18.8	2.6
13. Often unhappy, downhearted or tearful.	87.6	11.6	0.8	90.9	6.8	2.3	83.2	16.0	0.8	87.2	10.3	2.6	94.8	5.2	0
16. Nervous or clingy in new situation, easily lose confidence.	68.9	27.9	3.3	72.7	24.8	2.5	71.0	22.1	6.9	82.1	14.5	3.4	85.4	13.2	1.4
24. Many fears easily scared.	82.6	15.7	1.7	90.1	9.1	0.8	79.4	20.6	0.0	91.4	6.9	1.7	94.2	5.8	0
**Conduct problems items**															
5. Often has temper tantrum or hot tempers.	86.4	13.6	0.0	76.0	19.0	5.0	77.1	14.5	8.4	81.0	12.9	6.0	88.2	7.2	4.6
*7. Generally obedient, usually does what adults request.*	13.3	41.7	45.0	7.4	30.6	62.0	4.8	27.8	67.5	9.4	21.7	68.9	4.9	19.6	75.5
12. Often fights with other children or bullies them.	94.4	5.6	0.0	77.7	19.0	3.3	83.1	15.4	1.5	84.5	10.3	5.2	90.2	7.7	2.1
18. Often argumentative with adults	88.5	10.6	0.9	72.7	23.1	4.1	77.1	20.6	2.3	81.9	12.1	6.0	79.2	19.4	1.4
22. Can be spiteful to other	84.3	13.9	1.9	71.7	21.7	6.7	70.2	26.7	3.1	75.4	18.4	6.1	78.9	19.7	1.4
**Peer problems items**															
6. Rather solitary, tends to play alone.	59.3	34.1	6.5	67.9	26.7	5.3	75.6	16.0	8.4	81.2	17.1	1.7	85.6	14.4	0
*11. Has at least one good friend.*	28.8	43.2	27.9	8.4	20.6	71.0	2.3	9.2	88.5	3.4	8.6	87.9	3.8	6.4	98.7
*14. Generally liked by other children.*	18.5	29.0	52.4	3.8	19.2	76.9	2.3	19.1	78.6	0.9	22.2	76.9	1.9	10.1	88.0
19. Picked on or bullied by other children.	99.1	0.9	0.0	96.7	3.3	0.0	98.5	1.5	0.0	95.7	3.4	0.9	96.5	2.1	1.4
23. Gets on better with adults than with other children.	57.1	37.5	5.4	72.5	22.9	4.6	71.4	22.2	6.3	71.9	23.7	4.4	75.6	19.9	4.5

Children with another mother tongue than Swedish had no significant differences in the total difficulties scale, but this did not apply in two of the subscales. They got lower ratings on the Prosocial scale (*p* < .001) and higher ratings on the Peer problem scale (*p* = .009) compared to children with Swedish mother tongue. Children who were considered to be in need of special support had significantly higher total problem scores and higher ratings on all of the SDQ subscales (*p* < .005), except for the Emotional scale. They also received significantly lower ratings on the Prosocial scale (*p* < .001).

### Endorsement rates in different ages

#### Prosocial subscale

The response categories that clearly indicated low Prosocial behaviour skills were highest in the youngest children, diminishing as the age of the child increased. For children of 1 year of age, the alternative “not true” was more common on the items, “Shared readily with other children”, “Helpful if someone is hurt, upset of feeling ill”, and “Often volunteers to help others”.

#### Hyperactivity scale

Ratings of difficulties on the Hyperactivity subscale increased between the age of 2–3 years and then diminished between the age of 4–5 for the items 2, 10 and 15. Scores for the items “Can stop and think things out before acting” and “Sees tasks through to the end, good attention span” (reversed rating) increased progressively with growing age of the child.

#### Emotional subscale

All children received low ratings on items indicating emotional problems, the highest scores were found among 3-year old children on the item “Nervous or clingy in new situations, easily loses confidence”.

#### Conduct problem subscale

Scores for Conduct problems were similar among children in all ages except for the item “Generally obedient, usually does what adults request” (reversed rating), which had less endorsement for 1-year old children.

#### Peer problem subscale

For the Peer problem subscale the scores were generally low, except for the items “Has at least one good friend” and “Generally liked by other children”, that had less endorsements for the younger children.

### Endorsement rates on the supplement

The preschool teachers reported difficulties in the SDQ impact supplement in one or more of the areas: emotions, concentration, behaviour or being able to get on with other people, for 139 (17.1%) children.

Endorsements of the item” How long in months have these difficulties been present” increased with age. The difficulty had been present in “Over a year” for 29.5% of children of 2–3 years of age and 80% for children of 4–5 years of age.

Table 3 presents the ratings for the 139 children with an answer of “yes” on the SDQ supplement item “Overall, do you think that this child has difficulties in one or more of the following areas; emotions, concentration, behaviour or being able to get on with other people”. For the item “Do the difficulties upset or distress the child” endorsements of the alternative “Not at all” decreased by age from the youngest, 75%, to the oldest, 27%. The difficulties were judged to disrupt everyday life (free play, in organized situations, in routines) for children increasingly with rising age.

**Table 3:** Preschool teacher reports in the Supplement, 139 children with minor or severe difficulties aged 1- years (13–71 months). Endorsement rates (%) of response categories for each supplement item

SDQ Supplement	1 year				2–3 years				4–5 years			
	Not at all	Only a little	Quite a lot	A great deal	Not at all	Only a little	Quite a lot	A great deal	Not at all	Only a little	Quite a lot	A great deal
Do the difficulties upset or distress the child?	75	8.3	16.7	0	53.6	38.1	8.3	0	26.9	50	23.1	0
Do the difficulties interfere with the child’s everyday life in the following areas – free play	61.5	23.1	15.4	0	26.2	41.7	26.2	6	13.5	53.8	25	7.7
- organization	69.2	7.7	23.1	0	32.1	45.2	20.2	2.4	17	45.3	30.2	7.5
- routines	69.2	23.1	7.7	0	41.7	34.5	22.6	1.2	24.5	52.8	15.1	7.5
Do the difficulties put a burden on you or the class or group as a whole?	53.8	46.2	0	0	21.2	54.1	23.5	1.2	19.2	55.8	21.2	3.8

The preschool teachers rated that the difficulties put a burden on themselves or the class or group as a whole to a lesser degree in the youngest group, children of 1 year of age.

#### Total impact scale

In the whole sample the mean was 1, median 0, range 0–8, and mode 0. On the total impact scale, 17.0% of the children had a score of 1, 15.7% of the children had a score of 2, 13.9% scored 3, 9.6% scored 4, 6.3% scored 5, 3.3% scored 6 and 2.0% hade a score of 7–8. There were no significant differences between genders. It is important to identify children who are observed as having problems, so a score of 1 or above could be regarded as a cut off.

The correlation between SDQ total problems scale and total impact scale was *r* = 0.45, *p* < .01.

### Longitudinal analysis

**Fig. 1:**
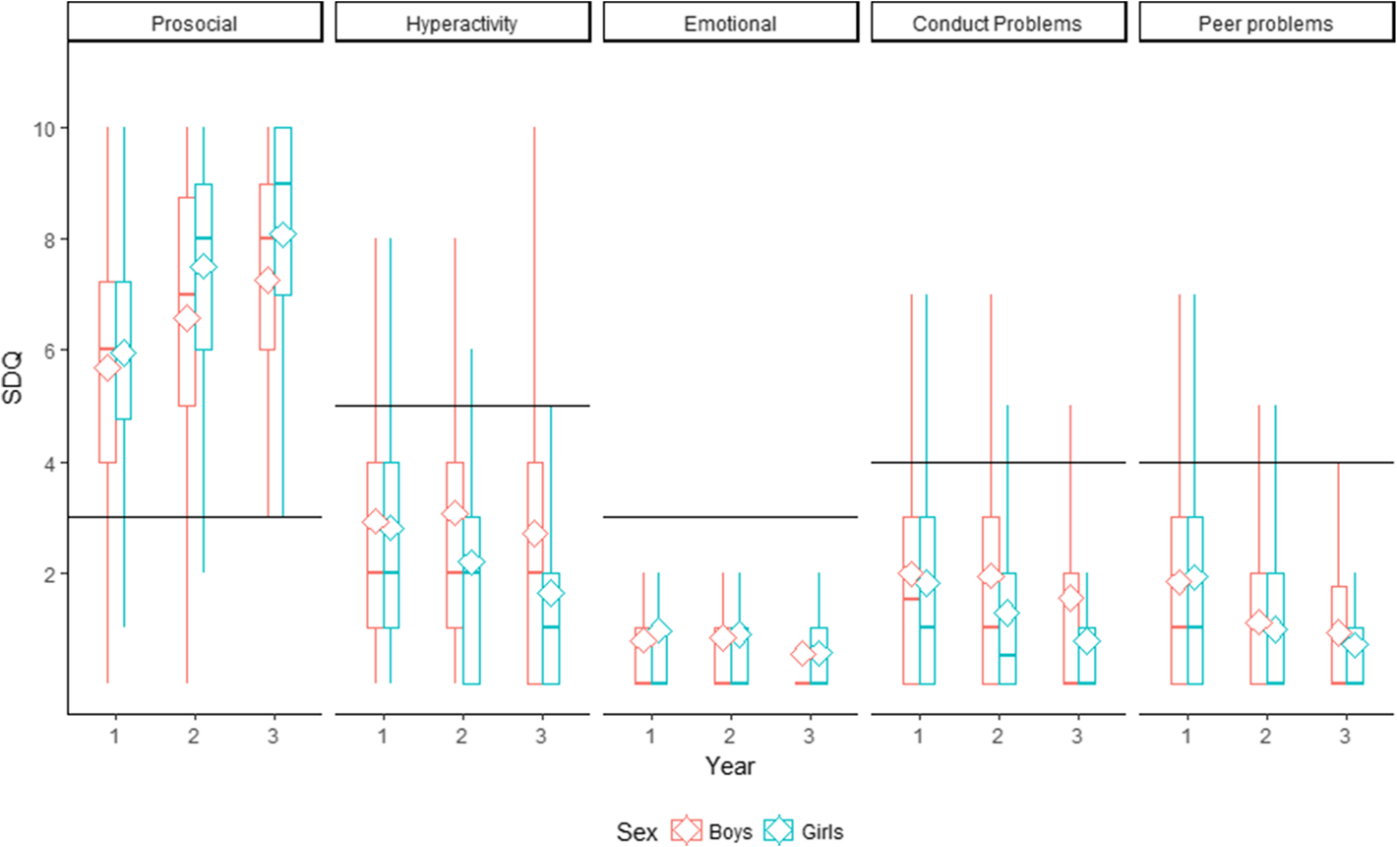
Box plots of the SDQ subscales longitudinally over three years, split by sex (boys in *red*). The boxes mark the 25th and the 75th percentile and the whiskers marks the highest/lowest value within 1.5 of the interquartile range from the box. The mean is marked with a rhomb, the median with a horizontal line and the cut-off whit a solid line

The scores on the SDQ subscales are presented longitudinally over three years, split by gender, in Fig. 1. Prosocial strengths increased over the years. Girls were constantly estimated as having less problems than boys. Regarding Hyperactivity, boys showed quite constant problem levels between years 1 and 2 while the ratings of Conduct problems increased. However, at time 3 both Hyperactivity and Conduct problems dropped quite markedly. Girl’s hyperactivity and conduct problems decreased in a constant way over time. For both boys and girls the ratings of Emotional symptoms were consistently low while Peer problems decreased over time.

## Discussion

Boys were reported to have significantly more problems than girls on the entire scale, and on all subscales except for the Emotional subscale. Boys were also considered to have significantly less Prosocial skills than girls. Thus gender differences seen in older children seem to be already present in preschool. Such gender differences were also found in other studies [11, 28, 41]. Perhaps this may indicate that different cut-off limits should be used for each gender.

Children who were considered to be in need of special support, ie. they were seen as problematic in the preschool group, had significant higher ratings on the whole scale and all the SDQ subscales, except for the Emotional scale. Thus, SDQ can be used for screening and identifying children with psychiatric symptoms early in life.

Children with first languages other than Swedish, i.e. born to immigrants parents, showed lower Prosocial ratings and higher ratings on the subscale Peer problems. It might be that these children have a problem with interpersonal communication to overcome, as reflected in these subscales [42]. It is tempting to propose that problems with the Swedish language might contribute to this. Looking at the different items, there is a progress in prevalence as the child grows older, with more prosocial behaviour and fewer behavioural problems the older the child becomes, except for the period between 2 and 3 years of age. This can be seen as a reflection of normal development. In the early stages, high motor activity is abundant, while play with same-age peers and prosocial capacity has not yet developed [42]. It is probably natural that small children do not have a best friend and, in the child group, it is not clear who is popular or not [43]. Nonetheless, it is important to identify bullying since this is a negative factor in the development of subsequent mental illness [44].

The proposed Swedish norms for SDQ 90th percentiles, based on this sample of Swedish preschool children rated by their teachers in a preschool setting, were similar to other European countries except for the Prosocial subscale, which had a lower score and the Conduct subscale, in which Swedish children were estimated to have more problems. More disruptive behaviour in Swedish preschools may be due to a preschool setting with more free play, and less organized situations. Less prosocial behaviour compared to European children was also shown by Heiervang et al. for Nordic school-age children (8–10 years) [16].

Goodman [45] declares that the best strategy for researchers is to choose cut-offs for the sample and the country being studied. Based on this idea, we recommend a rating of 12 on the SDQ Total problems scale as a cut-off for children in Swedish preschools, scored by preschool teachers. Our cut-offs on the various subscales match overall well with UK norms, and, on the total scale, our proposed cut-off is similar to the 11–14 rating proposed by Goodman [35]. Our Cut-off is also close to the score that Sim et al. found in children of 30 months of age as predictive for psychiatric problems 1–2 years later [5].

The overall prevalence shown in this study is similar to other industrialized countries [11, 46] but there are also studies showing higher problem scores from European countries, especially for the emotional scale, compared to the Nordic countries [16, 32, 41].

Studies using other screening instruments [17, 47, 48] have found noticeably lower prevalence’s of parent-reported symptoms and disorders among preschool and school age children in the Scandinavian countries compared to many other countries, including the US. There are also studies using SDQ that indicate lower scores among preschool children in the Scandinavian countries than in most other countries [17, 27].

Comparing the prevalence rates for behavioural problems with other studies from the Nordic countries shows similar results. Wichström et al. [27] in a study from Norway showed similar prevalence rates of behaviour problems using parents as informants, although fewer children in Norway seemed to have major problems (8% scoring over 11 on SDQ total scale) while, in this study, more children seem to have greater problem (15% with scores over 11). This may be due to using different informants and rating the behaviour in the preschool environment.

In a study by Ghaderi et al. from Sweden where parents rated their 2–5 year old children with SDQ [49], the cut-offs for the 90th percentiles were consistent with our findings for subscales, except that the parent’s ratings on the Prosocial subscale were slightly higher. Both studies show that behavioural problems can be already identified in young children.

In the Supplement, the mean Impact score was calculated as 1 in this study, as proposed by Goodman. This indicates that the child has some kind of difficulty that is apparent in everyday life [35]. Children with high scores on the SDQ problem scales also tended to have high scores on the Impact Supplement, though the statistical relationships are not always strong [33, 34]. Children may have high problem scores and still function well on a daily basis and vice versa, as shown in other studies [50]. The correlation between SDQ problem scales and supplementary questions increases with age. The SDQ Supplement also seem to capture some children with problems in everyday functioning that are not “found” if only SDQ problem scales are used to identify behavioural problems [34].

According to the supplement, the difficulties identified seems to distress the child more with increasing age and also disturb the group in different situations more as the child grows older. It might be that, as the child grows older, the problem both interferes with development and with social interaction.

Considering the longitudinal findings, boys showed quite constant levels regarding hyperactivity between year 1 and 2 while the ratings of conduct problems increased. At year 3 these externalizing behaviours dropped quite markedly. This could it be due to increasing socialization at the preschool with a better function the third year, however, it may also reflect increasing age. The instructions to the preschool teachers were to estimate the child’s behaviour according to what they considered appropriate for the child’s age.

Peer problems and conduct problems figures developed in a similarly pattern indicating a connection between these problem areas. Ratings of Prosocial behaviour increased over the three years, probably reflecting development of social skills.

The items on the Emotional scale where estimated low, not showing any developmental pattern over time. Further research is needed in this area, illuminating small children’s emotional problems and how to identify these children. Do the children small emotional problems, or is it that they are not detected, or detected late.

The present study has some limitations. A larger proportion of preschools from medium-sized municipalities and comparatively fewer preschools from large municipalities participated, compared to the population distribution in Sweden. This may have an impact on the prevalence rates. However, there is a Swedish study of school children of 12–16 years of age that shows that, regardless of size of the municipality, the proportion of children with major mental health problems was equally extensive [51]. There was quite a large drop-out; only 40% of the children attending the preschools were assessed. For ethical reasons, we required informed consent from both parents, which might have contributed to the rather low participation rate. There are no guarantees that the drop-out children have the same prevalence of problems. Using proxy informants is always difficult and with some risk for influencing findings. A large number of preschool teachers participated in the study and no evaluation of inter-rater reliability was made. The main strength is the considerably large sample that, in most areas, corresponds well with demographic data presented by the Swedish National Agency for Education [37] and might be regarded as representative for preschool children in Sweden. Furthermore, preschool staffs have a documented high level of understanding about child development [19].

Further research should be conducted on the development of children identified with SDQ early in life to see if they develop psychiatric problems. It is also important to see if SDQ can be used to evaluate interventions for children with identified problems.

Future research should also aim to develop validated methods to help children with early identified emotional distress/behavioural problems [32].

## Conclusions

The teacher version of the SDQ, for 2–4 year-olds, can be used as a screening instrument to identify early signs of emotional distress/behavioural problems in young children. Preschool teachers seem to be able to identify children with problematic behaviour with the use of SDQ at an early age. The development of behaviour over time differs for the different subscales of SDQ. The Swedish norms for SDQ are to a large extent, similar to findings from other European countries.

## Abbreviations

ADHD: Aattention Deficit Hyperactivity Disorder
CD: Conduct Disorder
DSM: Diagnostic and Statistical Manual of Mental Disorders
GAD: Generalized Anxiety Disorder
ICD: International statistical Classification of Diseases and related health problems
ODD: Oppositional Defiant Disorder
SAD: Separation Anxiety Disorder
SDQ: The Strengths and Difficulties Questionnaire
